# Extrahippocampal Seizure and Memory Circuits Overlap

**DOI:** 10.1523/ENEURO.0179-22.2022

**Published:** 2022-07-22

**Authors:** Aijaz Ahmad Naik, Anastasia Brodovskaya, Smriti Subedi, Amman Akram, Jaideep Kapur

**Affiliations:** 1 Department of Neurology, University of Virginia, Charlottesville, VA 22903; 2 College of Arts and Sciences, University of Virginia, Charlottesville, VA 22903; 3 University of Virginia Brain Institute, University of Virginia, Charlottesville, VA 22903; 4 Department of Neuroscience, University of Virginia, Charlottesville, VA 22903

**Keywords:** extrahippocampal, GluA1 KO, memory, retrograde amnesia, seizures, TRAP2

## Abstract

Seizures cause retrograde amnesia. We have previously demonstrated that seizures erode recently formed memories through shared ensembles and mechanisms in the CA1 region of the hippocampus. Here, we tested whether seizure circuits overlap spatial memory circuits outside of the CA. Spatial memory is consolidated by the hippocampal-cortical coupling that are connected via multiple pathways. We tested whether a seizure invades structures involved in memory consolidation by using the activity reporter TRAP2 mice. T-maze alternation learning activated neurons in the dentate gyrus (DG), mediodorsal thalamus (MD), retrosplenial cortex (RSC), and medial prefrontal cortex (mPFC). This spatial memory relies on the plasticity of the AMPA receptor GluA1 subunit. GluA1 knock-out (KO)/TRAP2 mice did not learn to alternate, and structures interposed between the hippocampus and the cortex were not active. A seizure prevented the recall of alternation memory and activated memory-labeled structures. There was a widespread overlap between learning-activated ensembles and seizure-activated neurons, which likely contributes to retrograde amnesia.

## Significance Statement

We propose that seizures cause retrograde amnesia by engaging the circuits that participate in memory consolidation.

## Introduction

Seizures cause retrograde amnesia, and the mechanisms are poorly understood. Seizures cause transient declarative memory deficits that occur even in patients who do not have underlying hippocampal pathology, in contrast to fixed epilepsy-memory deficits (Hoppe, 2007). Electroconvulsive therapy-induced seizures also cause an amnestic effect on recently acquired event memories (Duncan, 1949; Chorover and Schiller, 1965; Squire et al., 2001). We seek to understand the mechanisms of seizure-induced retrograde amnesia.

Consolidation of declarative memories requires hippocampal-neocortical communication (Khodagholy et al., 2017). The standard memory consolidation model proposes that recent memories fade from the hippocampus and get more robust in the neocortical modules, becoming hippocampus-independent (Buzsáki, 1996; Siapas and Wilson, 1998; Mcgaugh, 2000; Wiltgen et al., 2004; Liu et al., 2012; Preston and Eichenbaum, 2013; Squire et al., 2015; Tonegawa et al., 2015; Yonelinas et al., 2019). A pattern of selective cell activity in the hippocampus entrains other cortical brain regions. Strong anatomic and functional connections of the retrosplenial cortex (RSC), medial prefrontal cortex (mPFC), entorhinal cortex (EC), and mediodorsal thalamus (MD) with the hippocampus code for space and time and are implicated in consolidation (Leutgeb et al., 2005; Moser et al., 2008, 2017; Igarashi et al., 2014; Howard and Eichenbaum, 2015).

The parietal, RSC, anterior cingulate cortex (ACC), and mPFC display ripple oscillations (100–150 Hz) concurrent with hippocampal ripples, which are strengthened during sleep following learning (Khodagholy et al., 2017). Pathologic interictal epileptiform discharges (IEDs) in the hippocampus are correlated with decreased ripple occurrence in the mPFC (Gelinas et al., 2016). On the other hand, hippocampal IEDs evoke mPFC spindle oscillations (9–16 Hz) via induction of cortical DOWN state, which is characterized by δ waves associated with neuronal hyperpolarization in the deep cortical layers, possibly leading to consolidation deficits. These studies suggested that epileptiform discharges interrupt physiological mechanisms coupling the hippocampus to the cortex, which may impair cognition in patients with temporal lobe epilepsy.

Mapping seizure and declarative memory-activated neurons is an alternative approach to understanding seizure-induced amnesia. We mapped neurons engaged in storing memory and its retrieval using activity reporter mice (Josselyn and Tonegawa, 2020). Seizure networks can be mapped with a cellular resolution using activity reporter mice as well (Dabrowska et al., 2019; Adotevi et al., 2020; Brodovskaya et al., 2021). Seizures like memory often travel from the hippocampus to the cortex (Adotevi et al., 2020). We tested whether seizures activate the memory circuit to propagate.

We used TRAP2 mice that express a fluorescent protein under the immediate early gene promoter c-Fos (Tasaka et al., 2018) to tag spatial memory-activated neurons beyond the classical CA1 region permanently and in the extrahippocampal circuit. The T-maze spatial learning task is dependent on the plasticity of the GluA1 subunit of AMPA receptors. We used GluA1 knock-out (KO) mice crossed with TRAP2 mice to confirm that labeled activated neurons were specific to spatial memory and not just locomotion or spatial navigation. A seizure shortly after a mouse has learned to alternate for a reward on the T-maze degraded performance the following day (Naik et al., 2021). We tested whether seizure-activated neuronal ensembles overlapped spatial memory-activated neurons by performing TRAP2 and ARC dual-labeling. We propose that seizures cause retrograde amnesia by engaging the circuits of memory consolidation.

## Materials and Methods

### Animal model

All studies were performed following the approved protocols of the Animal Care and Use Committee. For tagging activated neuronal ensembles following learning, we used TRAP2 mice generated by crossing mice expressing tdTomato from the Rosa locus [(B6.Cg-Gt(ROSA)26Sor^tm9(CAG-tdTomato)Hze^/J, ME, #007909; The Jackson Laboratory] to mice expressing Cre-ER under cFos promoter (Fos^2A-iCreER^, #030323; Tasaka et al., 2018). AMPA receptor KO mice (GluA1 global KO) were obtained from Seeburg (Max-Plank Institute for Medical Research, Heidelberg, Germany) and have been characterized in detail previously (Reisel et al., 2002; Adotevi et al., 2020). To permanently label the cFos-expressing neurons following learning in GluA1 KO mice, we crossed GluA1 global KO mice with TRAP2 mice to generate GluA1 KO/TRAP2 mice. Mice of both sexes were maintained on 12 h light (6 A.M. to 6 P.M.)/12 h dark (6 P.M. to 6 A.M.) cycle in a homecage (12 × 6 × 5 in) with corn cob bedding and had *ad libitum* access to food and water. For genotyping, KAPA Biosystems kit was used.

### Delayed discrete trial rewarded alternation task in T-maze

We trained TRAP2 mice (six to eight weeks, *n* = 40) individually housed to alternate on a T-maze for a reward of sweetened condensed milk. We food-deprived mice to no less than 80% of their beginning body weight a week before to motivate them for the reward. After a week of food restriction and familiarization with sweetened milk, we trained mice in a delayed discrete trial rewarded alternation task for 5 d (Naik et al., 2021). Days 1 and 2 served as a training session wherein no delay was introduced between sample and choice runs; however, on day 3 and afterward, a delay of 20 s was introduced between sample and choice trials. Percent correct choices were recorded for each mouse and also sample and choice latencies.

To confirm the behavioral phenotype of hyperactivity in the GluA1 KO mice, we tested the GluA1 KO/TRAP2 and littermate controls (TRAP2) in an Open Field Activity Monitor (Nodulus Instruments). The mice were individually tested for 20 min, and activity maps were used for phenotyping. This behavioral phenotyping was used in addition to the genotypic characterization of the GluA1 KO mice. Data are presented as heatmaps of the activity in the Open Field Activity Monitor.

### Labeling training activated neuronal ensembles

To permanently label the activated population of neurons following training and learning in the T-maze task, separate cohorts of TRAP2 mice were injected with fast-acting 4-hydroxy tamoxifen (4-OHT) 60 min after the training on days 1–3. We imaged learning-activated neuronal ensembles in CA1, dentate gyrus (DG), and RSC regions across multiple training sessions. Separate cohorts of mice were injected with 4-OHT on days 1–3. To avoid transfer of handling-induced neuronal activation, the mice were returned to home cages only an hour after 4-OHT injection. Mice were transcardially perfused with 4% paraformaldehyde (PFA) in 0.1 m PBS a week after 4-OHT injections and stored overnight in 4% PFA. The brains were cut coronally (40 μm), stained with DAPI or NeuN, and imaged at 10×/0.45 NA magnification on a Nikon Eclipse Ti2 fully motorized confocal microscope. Imaris version 9.8.0 (Bitplane Scientific) was used to create composite images. Unbiased manual counting of tdT^+ve^ neurons was done individually by two people blinded to the treatment in every slice. tdT^+ve^ neurons with well-defined cell bodies and processes in the granule cell layer of DG were counted only. Scrolling over the whole Z- stack was performed to avoid missing any tagged neurons. Fiji ImageJ was used for automated counting of tdT^+ve^ neurons in RSC, mPFC, MD, and paraventricular thalamus (PVT). Reference atlases from Allen as well as Paxinos were used to define the boundaries of the target brain regions (DG, mPFC, RSC, PVT, and MD) in coronal slices.

### TRAPing seizure-activated neuronal populations

TRAP2 mice (six to eight weeks old) were injected with PTZ (40 mg/kg, i.p.) and observed for a generalized tonic-clonic seizure. Mice that experienced a seizure were injected with 4- OHT 60 min after the seizure. Mice that received saline injections served as a control. All mice injected with 4-OHT were perfused after a week and processed for imaging as mentioned above. Tagged neuronal populations following a seizure were counted in the brain regions like DG, RSC, mPFC, MD, PVT in every slice, and presented as mean ± SEM.

### Dual-labeling of neuronal ensembles activated following rewarded alternation learning and seizure

First, using 4-OHT injections on day 2 of the T-maze, the learning-activated neuronal ensembles were permanently tagged with tdTomato. The same mouse received a PTZ injection a week later to cause a seizure. Mice were killed 60 min after the seizure. Arc immunohistochemistry was performed using the antibody (1:2000, Rb polyclonal, 156003, Synaptic Systems). Dual-labeling results were confirmed and validated using an anti-cFos antibody (1:1000, Rb polyclonal, ab190289, Abcam) as well in TRAP2 mice. Those mice that were injected with 4-OHT in a home-cage and later given a seizure served as controls for the dual-labeling experiment. To test whether the observed overlap exceeded the probability of random overlap, we conducted the following calculation (Roy et al., 2017): overlap probability = ((day 2 learning-activated tdT^+ve^ neurons in a region/Total DAPI^+ve^ neurons in that region) × (seizure-activated tdT^+ve^ neurons in the same region/Total DAPI^+ve^ neurons in that region)) × 100%.

### Statistical analysis

Data are expressed as means ± SEM. Graphs were prepared in Adobe Photoshop CC, images were created with BioRender.com, and statistical analysis was done using GraphPad Prism 8.0 software (GraphPad Software Inc.). Two-tailed Student’s *t* tests were used to compare saline and seizure groups. Multiple *t* tests were used for comparisons among two treatment groups with multiple brain regions under analysis. One-way ANOVA was used for multiple group comparisons. Black asterisks in figures indicate significance by Student’s *t* test. Data were tested for normality before the use of *t* tests and multiple comparison tests.

## Results

**Figure 1.**
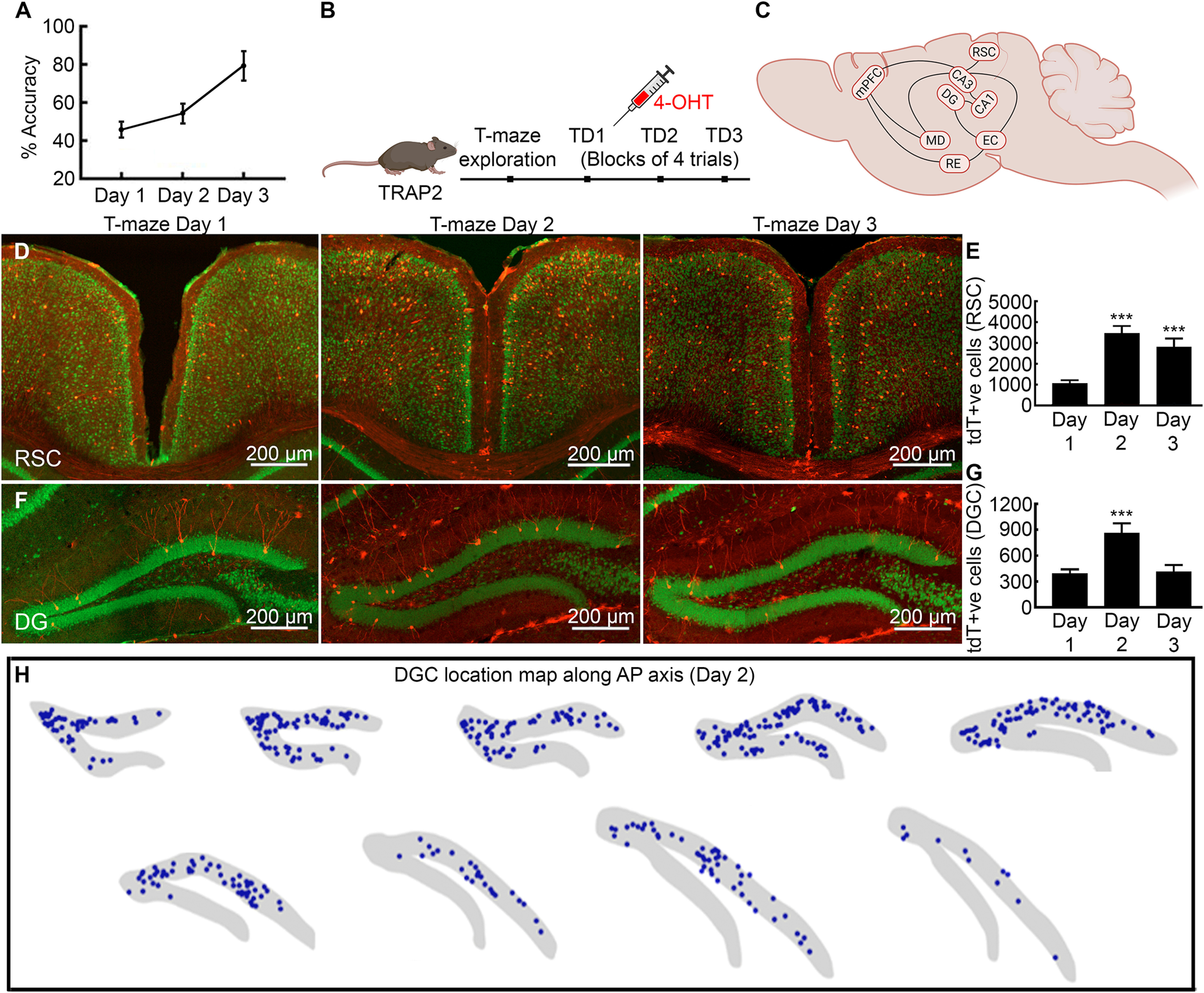
Rewarded alternation task learning triggers the formation of hippocampal and cortical ensembles. ***A***, TRAP2 mice learn to alternate for reward on day 2 and show successful alternation with high accuracy on day 3. ***B***, A schematic of the experimental design for tagging learning activated neuronal ensembles following spatial memory task. ***C***, A hypothetical circuit diagram for spatial memory engram of brain structures with anatomic connectivity and established role in spatial memory. ***D***, Activity-dependent neuronal activation following T-maze training in the RSC on days 1–3 with tdTomato+ve neurons in layers 2/3 and 5 of both granular and dysgranular regions. ***E***, A significantly larger fraction of RSC neurons was tagged on day 2 and many remained active on day 3. ***F***, Learning activated DGCs across training sessions from day 1 to 3. A large fraction of DGCs was tagged on day 2, whereas by day 3, the number dropped. ***G***, Mean number of DGCs labeled on day 2 compared with day 1. ***H***, Location maps show DGCs activation on T-maze training day 2 across the anterior-posterior axis (blue dots represent every tagged DGC). Data presented as mean ± SEM, ***p < 0.001, ANOVA and *post hoc* Tukey’s test.

We trained activity reporter TRAP2 mice to alternate on a T-maze for a reward to map neuronal ensembles recruited while learning a spatial memory task. On the second day of training, mice acquired the rewarded alteration memory because they demonstrated improved performance on the following and subsequent days (Fig. 1*A*; Naik et al., 2021). Therefore, memory encoding occurred on day 2, and memory retrieval occurred on day 3. To tag and identify learning-activated neuronal ensembles, we injected separate cohorts of TRAP2 mice with 4-OHT on training days 1–3, 60 min after training (Fig. 1*B*). We selected 60 min because c-Fos mRNA expression peaks during that time (Dabrowska et al., 2019). Previous studies find CA1 pyramidal neuron activation during this spatial memory task (Naik et al., 2021). Here, we tested the activation of neurons in the structures outside of the CA1 and extrahippocampal systems that receive projections from the CA1 (Fig. 1*C*).

### Dentate recruitment

We first analyzed spatial memory-activated neurons in the DG that plays a role in pattern separation and forwards the information to the CA1 region of the hippocampus. The acquisition and retrieval of episodic memories engage dentate granule cells (DGCs; Liu et al., 2012; Tonegawa et al., 2015; Josselyn and Tonegawa, 2020). After T-maze training, we counted tagged DGC neurons manually and by an automated counting method in every slice and found a two-fold increase on day 2 in the number of tagged DGCs than on the previous day (Fig. 1*F*,*G*, day 1: 390.0 ± 51.23; day 2: 859.0 ± 114.90; day 3: 411.3 ± 78.22, one-way ANOVA, *n* = 6 mice, *p* = 0.0063). The density of tagged DGCs was higher in anterior dorsal than posterior ventral regions (Fig. 1*H*). Neurons were clustered near the hinge joining the supra-pyramidal and infra-pyramidal blades. There were more tagged DGCs in the supra-pyramidal than in the infra-pyramidal blade. The dorsal slices had tagged DGCs in both supra-pyramidal and infra-pyramidal blades, whereas the ventral slices had tagged DGCs only in the supra-blade (Fig. 1*F*,*G*). Consistent with the standard model of memory consolidation, day 3 group had fewer labeled DGCs compared with the previous day (Fig. 1*G*, *p* = 0.019). The CA1 pyramidal neuronal recruitment follows a similar trend across training sessions (Naik et al., 2021).

### Learning-activated RSC ensembles

The RSC is positioned at the interface between hippocampal formation and cortical sensory regions and receives direct input from CA1 and subiculum (Sugar et al., 2011). This connectivity makes it a candidate for encoding and retrieving spatial memory (Todd et al., 2019; Vann et al., 2009). We found a threefold increase in tagged neuron population on day 2 group compared with day 1 of training (Fig. 1*D*,*E*, day 1, 1069.0 ± 146.60, day 2, 3466.0 ± 356.20, day 3, 2808.0 ± 414.0, one-way ANOVA, *n* = 6, *p* < 0.05, *F* = 11.07). Labeled neurons in RSC were mainly seen in layers 2/3 and 5 of granular and dysgranular regions and crossed the rostrocaudal axis (Fig. 1*D*). The dorsal part of the RSC had a higher density of tagged cells than the ventral. On day 3, the RSC was as active as observed on day 2 (Fig. 1*E*).

### The PFC and MD

**Figure 2.**
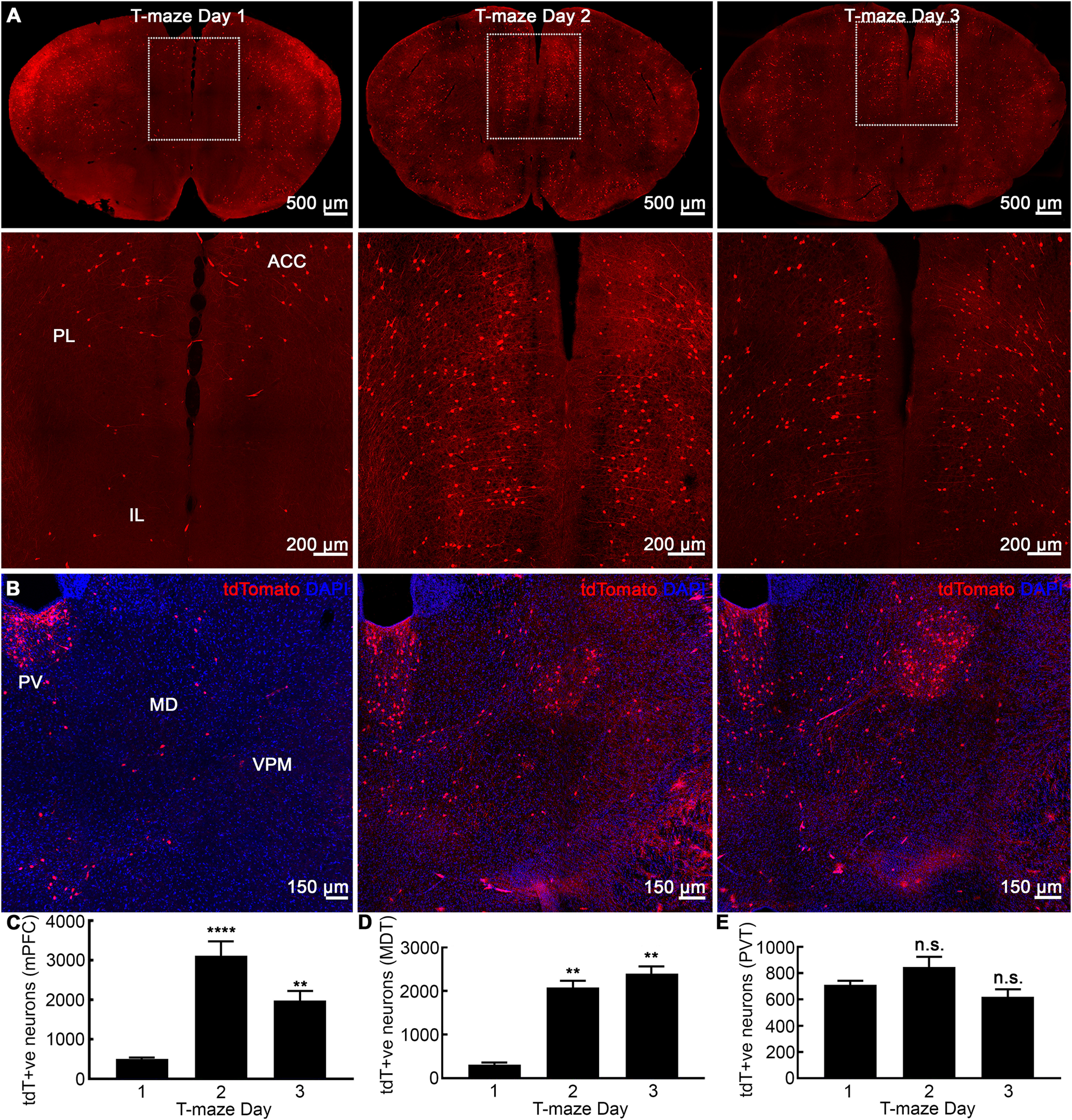
Activation of cortical modules in a working memory paradigm. ***A***, Representative coronal sections showing learning-tagged neuronal ensembles in the mPFC, which consists of PL, ILA, and ACC cortices across training days 1–3. There were more tagged neurons on days 2 and 3 compared with day 1. ***B***, Coronal sections through the thalamus with tdTomato (red) and DAPI (blue) staining. On days 2 and 3, learning-triggered neuronal ensembles were evident in MD and LD, which were absent on the first day of training. As a control structure, the PVT was quiescent with no changes across the training sessions. ***C***, Number of tagged neuronal ensembles in mPFC showing a 7- and 5-fold increase on days 2 and 3 compared with day 1. ***D***, MD had many learning-tagged neurons on days 2 and 3. ***E***, Labeled neuronal population in PVT remained unchanged across days 1–3. Data are mean ± SEM, *n* = 6 per group, **p < 0.01, ****p < 0.0001, ANOVA *post hoc* Tukey's test.

We then assessed whether rewarded alternation learning activated neurons in the dorsal mPFC, which consists of prelimbic (PL), infra-limbic (IL), and ACC and receives direct projections from the hippocampus (Gelinas et al., 2016). Compared with day 1, the fraction of tdTomato+ve neurons in mPFC was significantly more the following day (Fig. 2*A*,*C*, day 1, 508.33 ± 30.41, day 2, 3127.20 ± 351.23, day 3, 1977.33 ± 246.28, one-way ANOVA, *n* = 6, *p* < 0.0001, *F* = 30.19). There were learning-tagged mPFC neuronal ensembles on day 3 with similar recruitment levels as observed on day 2 (Fig. 2*A*,*C*).

The MD thalamus has extensive reciprocal connections with the mPFC (Mitchell and Chakraborty, 2013), amygdala (Kim and Cho, 2017), and EC (Yang et al., 2019), all of which receive projections from the CA1. Optogenetic inhibition of MD terminals that project to mPFC impairs T-maze task performance (Mitchell and Chakraborty, 2013; Saalmann, 2014; Bolkan et al., 2017). We found very few labeled neurons in MD on day 1 and a larger labeled population on the second day (Fig. 2*B*,*D*). Interestingly, during the retrieval on day 3, the MD was as active as on day 2 (Fig. 2*B*,*D*). We also studied the PVT as a control region with no role in spatial learning/memory (Fig. 2*B*,*E*). We did not find any differences in tdTomato+ve neuronal population in PVT across training days 1–3 of rewarded alternation task (Fig. 2*B*,*E*).

### Poor recruitment of hippocampal/cortical neuronal ensembles in GluA1 KO mice

We then confirmed that neuronal activation during the T-maze task was specific to the spatial working memory neuronal activation and not just because of the locomotion or spatial navigation during the task. The T-maze spatial working memory task is dependent on the plasticity of the GluA1 subunit of AMPA receptors (Sanderson et al., 2008). Mice that lack GluA1 subunit have impaired spatial working memory that is GluA1-dependent, short-term memory. In contrast, these mice show normal spatial reference memory acquisition such as hidden-platform water-maze task that requires GluA1-independent, long-term memory (Reisel et al., 2002; Sanderson et al., 2008).

**Figure 3.**
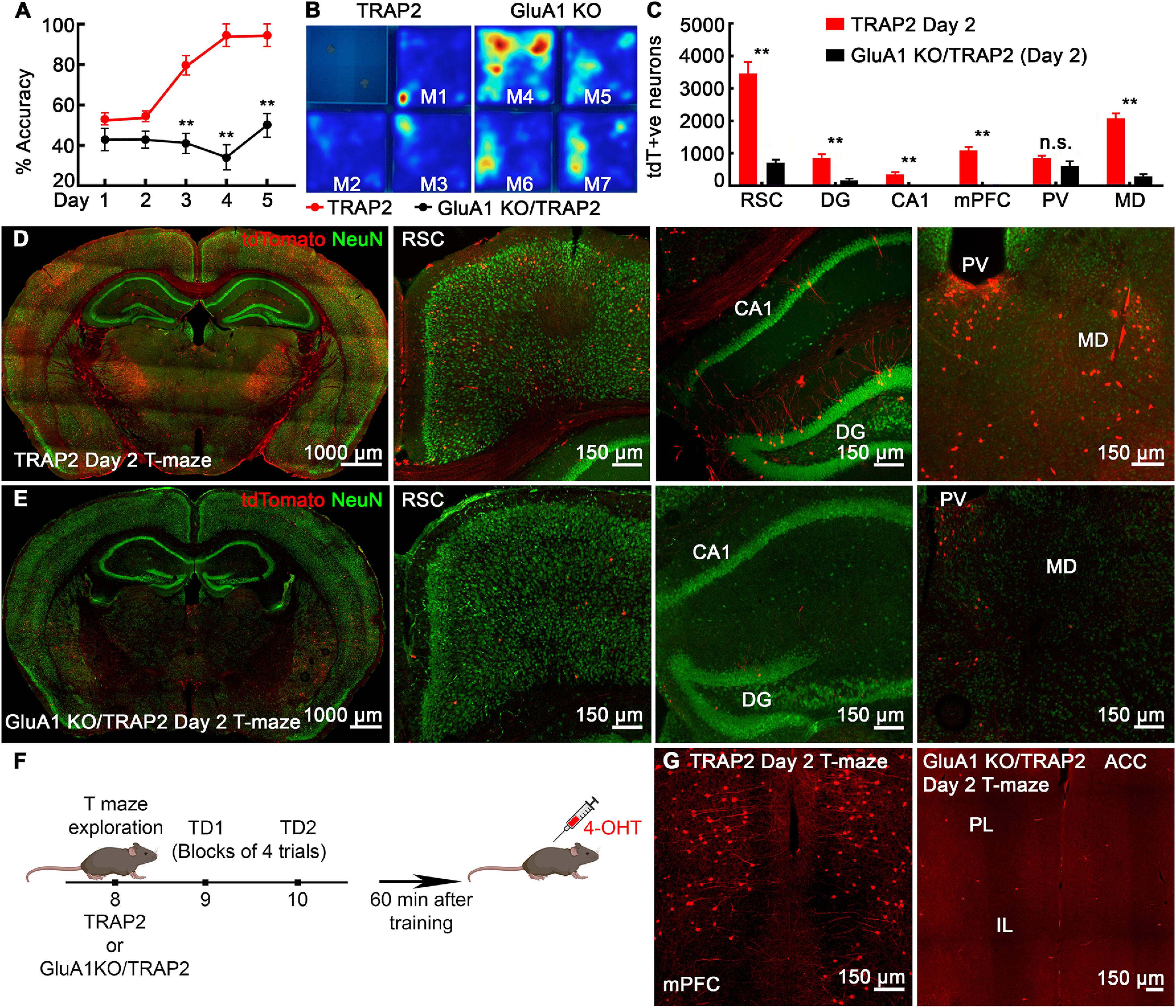
GluA1 KO/TRAP2 mice fail to learn rewarded alternation and show poor recruitment of memory ensembles. ***A***, Learning curve for mice in the T-maze task shows GluA1 KO/TRAP2 mice fail to learn to alternate for reward even after prolonged training, whereas littermate TRAP2 controls show a normal learning curve. ***B***, Heatmaps of activity-monitoring in open field test (test session of 15 min) in control TRAP2 mice (mouse 1–3) and GluA1 KO mice (mouse 4–7) confirm hyperactivity phenotype in GluA1 KO/TRAP2 mice. ***C***, Quantification shows a significantly fewer number of learning-tagged neurons in GluA1 KO mice in RSC, DG, CA1, mPFC, and MD, but normal engagement in PVT compared with control TRAP2 mice. ***D***, ***E***, Confocal images show representative coronal slices (40 μm) at the level of the hippocampus with tdTomato (red) and NeuN (green) labeling from T-maze day 2 TRAP2 group mice (***D***) and T-maze day 2 GluA1 KO/TRAP2 group (***E***). Magnified images show more spatial memory-activated neurons on day 2 in TRAP2 controls than in GluA1 KO/TRAP2 mice in the RSC, hippocampus (CA1, DG), and MD thalamic nucleus. ***F***, A schematic of the experimental design. ***G***, mPFC is not engaged following learning on day 2 of T-maze in mice lacking GluA1. Data are presented as mean ± SEM, *n* = 6 mice for each group, **p < 0.01, *t* test for each pair TRAP2 controls versus GluA1 KO/TRAP2.

We tested global GluA1 KO mice for discrete delayed trial rewarded alternation task in T-maze and found that these mice did not learn to alternate. The accuracy rates of successful alternation for these mice were at the chance levels for all training days (see the learning curve; Fig. 3*A*). The littermate controls showed a typical learning curve for the task with alternation accuracy rates of above 75% by day 3 and stable expression on the following days of probe testing. When tested in the open field apparatus, the GluA1 KO mice were hyperactive independent of sex with no signs of habituation (M4–M7; Fig. 3*B*). In contrast, control mice habituated shortly after exploring novel environment (M1–M3; Fig. 3*B*).

We next determined neuronal ensembles recruited following training in GluA1 KO mice. To visualize activated neurons in GluA1 KO mice, we crossed global GluA1 KO with TRAP2 to create GluA1 KO/TRAP2 mice. GluA1 KO/TRAP mice were injected with 4-OHT 60 min after training on day 2 to tag the activated population following learning in T-maze (Fig. 3*F*). Visual examination revealed far fewer labeled neurons in these mice on day 2 than the TRAP2 controls (Fig. 3*D*,*E*). The hippocampus of GluA1 KO/TRAP2 mice showed significantly low recruitment of neuronal ensembles, with hardly two or three tagged pyramidal neurons in the entire CA1 compared with littermate TRAP2 controls (Fig. 3*C–E*). Also, far fewer DGCs were labeled in these mice on the second training day than in controls (Fig. 3*C–E*). There was a reduced engagement of RSC neuronal ensembles in GluA1 KO/TRAP2 mice (Fig. 3*C–E*, *n* = 6, *p* < 0.001). The mPFC of these mice was also devoid of any tagged neurons (Fig. 3*G*). There was poor recruitment of MD compared with controls (Fig. 3*C–E*). However, in contrast to this scarce global engagement, the tagged neuronal population in the PVT of GluA1 KO mice was similar to that in controls (Fig. 3*C–E*). Thus, failure to learn the alternation rule in GluA1 KO mice was associated with poor recruitment of memory ensembles in these crucial hippocampal and cortical sites otherwise engaged in TRAP2 littermate controls.

### Seizure network

**Figure 4.**
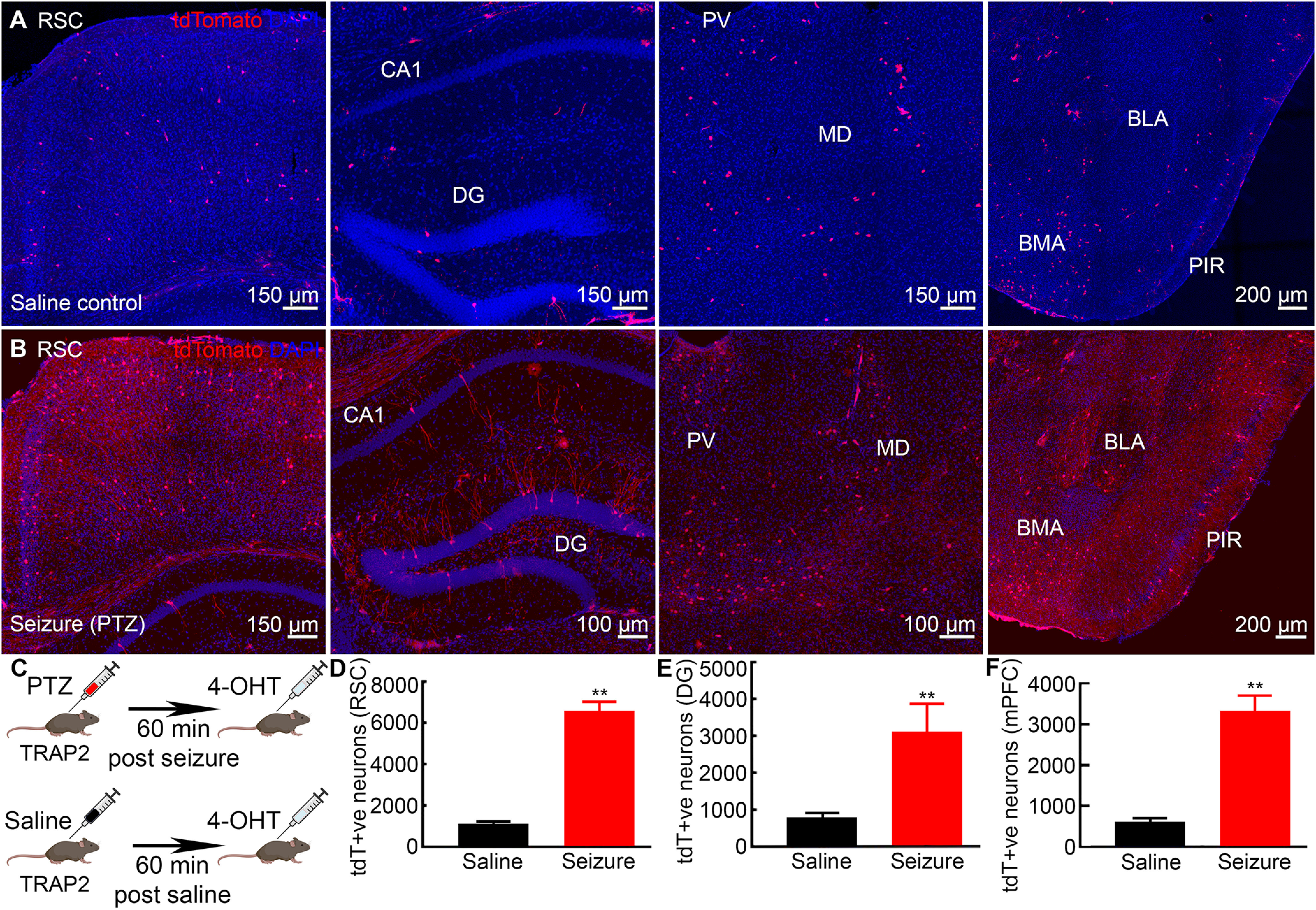
Neuronal network activated and engaged by a single seizure. ***A***, Representative images from the saline-treated group showing few tagged neurons in the RSC, hippocampus (CA1 and DG), MD, PVT, BLA, and BMA. ***B***, Generalized tonic-clonic seizure activated larger neuronal ensembles in RSC, hippocampus, thalamus as well as in amygdala. ***C***, A schematic of experimental design for tagging seizure-activated neurons. ***D–F***, A larger number of tagged neuronal populations was found in seizure-treated than saline-treated mice in RSC, DG, and mPFC. Data are presented as mean ± SEM, *n* = 6 mice for each group, **p < 0.01, Student’s *t* test.

Previous studies show that a single generalized tonic-clonic seizure immediately after day 2 of training-induced transient retrograde amnesia and impaired T-maze performance accuracy on day 3 (Naik et al., 2021). We tested whether a seizure also engaged memory circuits outside of the CA1 by TRAPing neurons (Fig. 4). Two investigators blinded to the treatment performed unbiased quantification of tagged neurons following either a seizure (PTZ, 40 mg/kg) or saline in TRAP2 mice across brain structures activated during spatial memory formation on day 2 (Fig. 1*C*). Visual examination showed a larger population of neurons labeled in mice that experienced a seizure than in saline controls (Fig. 4). There were significantly more tagged DGCs in the DG of mice that experienced a seizure (Fig. 4*B*,*E*, saline, 790.0 ± 119.3, seizure, 3091.0 ± 768.70, *t* test, *n* = 6 mice each, *p* = 0.018) than saline controls (Fig. 4*A*,*E*). Both infra-blade and supra-blade of the DG of the seizure group mice had many tdT^+ve^ granule cells across anterior-posterior axis.

We quantified seizure-activated tagged neuronal populations in RSC and mPFC. A single seizure tagged more neurons in the RSC than saline controls (Fig. 4*B*,*D*, saline, 1120.0 ± 106.20, seizure, 6560.0 ± 450.00, *n* = 6, *t* test, *p* < 0.01). Tagged neurons were present in layers 2/3 and 5 in both dorsal and ventral parts of the RSC in seizure-treated mice (Fig. 4*B*). There were tagged neurons across the anterior-posterior axis that involved both granular and dysgranular parts of the RSC. In addition to RSC, we found significantly more tagged neurons in the mPFC following a single seizure (Fig. 4*F*). Besides, a qualitative examination showed a large fraction of seizure-tagged neurons in brain regions like the piriform cortex (PIR), MD, EC, amygdala [basolateral amygdala (BLA), baso-medial amygdala (BMA)], and others (Fig. 4*B*) compared with saline controls (Fig. 3*A*).

### Seizure stimulates spatial memory-activated neurons

**Figure 5.**
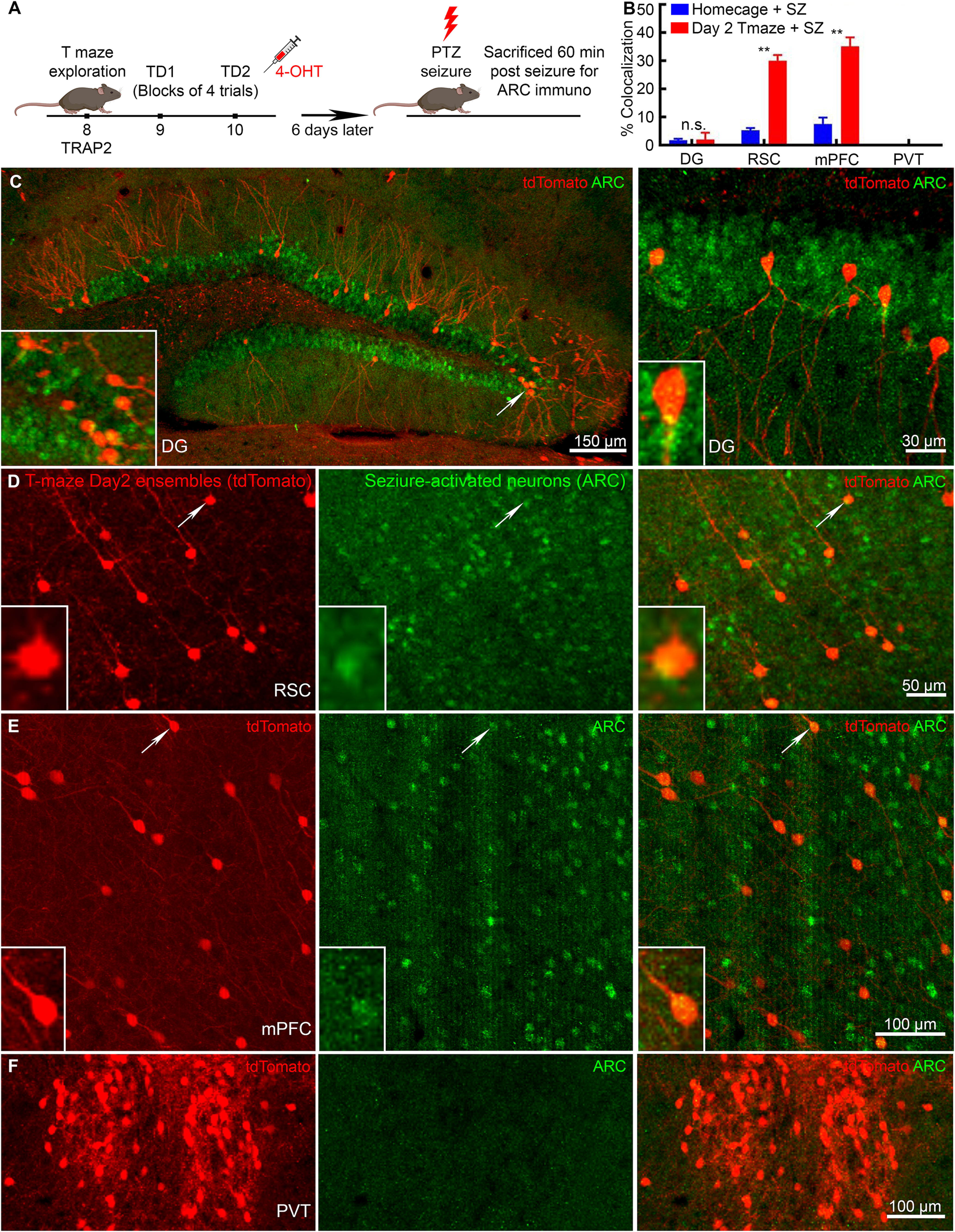
Memory and seizure-activated neuronal ensembles overlap in the neocortex but not in DG. ***A***, A schematic of experimental design for dual-labeling memory-activated and seizure-activated neurons in the same mouse. Mice that were injected with 4-OHT in homecage and later received a PTZ seizure served as control for dual-labeling experiment. ***B***, Co-localization of memory and seizure activated neurons in DG, RSC, mPFC, and PVT regions with high overlap in mPFC and RSC and no overlap in DG and PVT. ***C***, Memory-activated neuronal ensembles permanently tagged with tdTomato (red) and seizure-activated ARC-immunolabeled neurons (green) in DGC showed low levels of overlap among the ensembles. ***D***, Active neuronal ensembles in the RSC on day 2 (tdTomato) and seizure-tagged neurons (ARC) showed overlap. ***E***, Representative images of mPFC neurons labeled on day 2 of training (tdTomato) and seizure-tagged neurons (ARC) showed a large fraction of tdT^+ve^ neurons co-labeled for ARC. ***F***, In PVT, day 2 training- activated (tdTomato) and seizure-activated (ARC) neuronal ensembles did not overlap. Data are mean ± SEM, *n* = 6 mice for day 2 with seizures, *n* = 3 for home-cage with seizure, **p < 0.01, *t* test for each pair.

We next tested whether seizure-activated neuronal ensembles overlap spatial learning-activated neurons by performing dual-labeling. We first permanently tagged the learning-activated neurons in TRAP2 mice on day 2 of training, and a week later, the same mice received a seizure. The mice were killed 1 h after the seizure to detect Arc-labeled neurons (see the experimental design in Fig. 5*A*). The control mice were injected with 4-OHT in a home-cage to tag baseline neuronal activation and later received a seizure.

**Figure 6.**
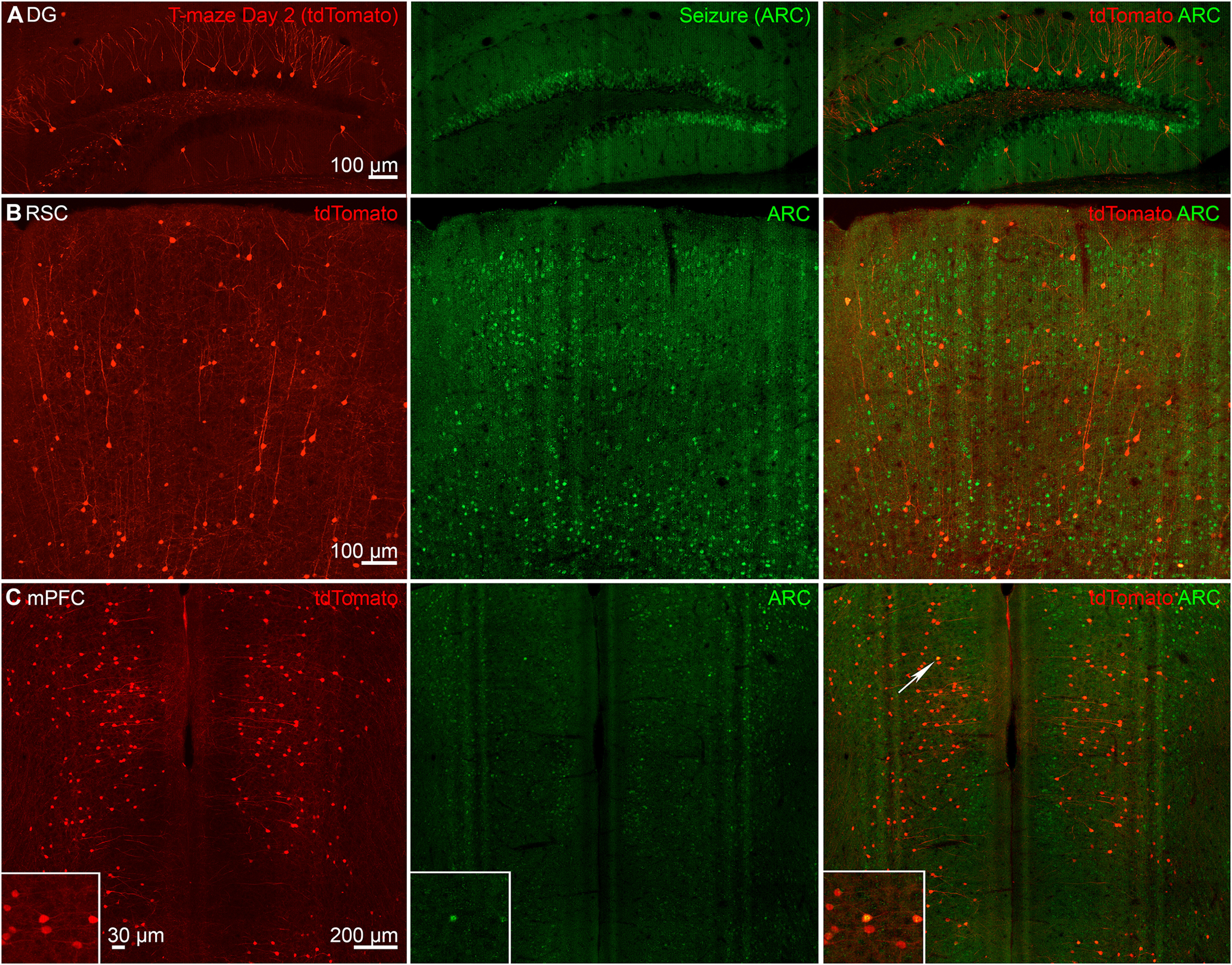
Cortical-neuronal ensembles activated following learning and seizure overlap. Representative images show learning-tagged neurons (tdTomato+ve, red) on day 2 of T-maze in (***A***) DG, (***B***) mPFC, and (***C***) RSC. ARC+ve neurons represent neuronal population activated following a single tonic-clonic seizure showed low levels of co-localization in DGCs, whereas mPFC and RSC showed higher levels of overlap between learning-tagged and seizure-tagged neuronal ensembles.

Only 2% of learning-tagged DGCs expressed seizure-activated ARC (Figs. 5*B*,*C*, 6*A*). The control mice showed a similar overlap (Fig. 5*B*), which could be a result of the basal activity in the DG. We also calculated joint probability of overlap for double labeling of tdTomato and ARC (overlap probability = ((day 2 learning-activated tdT^+ve^ neurons in a region/Total DAPI^+ve^ neurons in that region) × (seizure-activated tdT^+ve^ neurons in in the same region/Total DAPI^+ve^ neurons in that region)) × 100%). The probability of random overlap for DG was 0.0011% [((859/501 240) × (3091/501 240)) × 100%; Keller et al., 2018]. We next studied the extra-hippocampal structures, the RSC, and mPFC.

We found a 33% overlap between learning-activated neuronal ensembles and seizure-tagged neurons in the RSC (Fig. 5*B*,*D*). We observed co-labeled neurons in layers 2/3 and 5 of the dorsal RSC (Fig. 6*B*). This overlap in RSC in mice following day 2 training and a seizure a week later was significantly higher than observed in control mice (Fig. 5*B*). The probability overlap for RSC was 0.23% [((3466/98,148) × (6560/98,148)) × 100%; Keller et al., 2018; Murakami et al., 2018]. We also found a 35% overlap among memory and seizure tagged neurons in the mPFC, which was significantly larger than in the control mice (Fig. 5*B*,*E*; S1C). In contrast, the probability overlap was 0.070% [((3127/121,000) × (3255/121,000)) × 100%; Keller et al., 2018; Murakami et al., 2018]. Interestingly, in the control brain region (PVT) with no apparent spatial memory role, we did not find any overlap among the seizure and learning-activated ensembles (Fig. 5*B*,*F*).

## Discussion

We find that seizures and spatial learning activate the overlapping set of neurons widely distributed in the brain beyond the hippocampal subfields, causing retrograde amnesia. We mapped for the first-time neurons activated by spatial memory using activity reporter mice and showed their representation beyond the classical hippocampal CA1 in the brain regions like RSC, MD, and mPFC on the cellular level. The T-maze spatial learning task is dependent on the plasticity of the GluA1 subunit of AMPA receptors (Sanderson et al., 2008), so we used GluA1 KO mice crossed to TRAP2 mice to test whether widespread neuronal activation was specific to the spatial memory task and not because of the locomotion or spatial navigation. GluA1 KO/TRAP2 mice failed to learn the spatial memory task despite extended training sessions and lacked tagged neurons in the RSC, MD, and mPFC. A single seizure prevented the recall of alternation memory (Naik et al., 2021). Previous work demonstrated that seizures invade the circuits of memory-activated neurons in the CA1 region of the hippocampus, altering their physiology through saturated LTP (Naik et al., 2021). In this study, seizure-activated and spatial learning-activated ensembles also overlap in the regions outside of the CA1 and cortical structures, causing the failure of hippocampal-cortical interaction and the perturbation of systems consolidation.

We hypothesize that anatomic connectivity, together with neuronal excitability, inhibitory surround, and neuronal plasticity, shape the seizure circuit. The pattern of neuronal activation during seizures might be a stochastic process guided by the increased plasticity of certain cells. Neuronal plasticity level could increase or decrease the probability of cells being recruited by a seizure. Our previous work demonstrated that global removal of the GluA1 subunit of AMPA receptor reduced susceptibility to status epilepticus (Adotevi et al., 2020). GluA1 KO reduced mortality, severity, and duration of status epilepticus. This indicates that multiple plasticity mechanisms are critical for the occurrence and maintenance of seizures. Seizure-activated neurons are more excitable and have potentiated synapses (Naik et al., 2021). Synaptic connectivity and neuronal excitability optimize the efficacy of memory retrieval, contributing to memory formation and consolidation (Pignatelli et al., 2019). In addition, frontal lobe seizures follow its anatomic projections through the basal ganglia, specifically through the indirect pathway, the neurons of which are more excitable than those of the direct pathway (Brodovskaya et al., 2021).

The inhibitory tone might also play a role in shaping the seizure circuit. During kindling, neuronal firing and synchrony are more correlated along the lamellar hippocampal axis that contains excitatory connections than along the septotemporal axis that has inhibitory connections (Ren et al., 2021). Propagation of seizures through the hippocampus is gated by strong inhibition of the DG (Krook-Magnuson et al., 2015). Similarly, in the neocortex, the inhibitory surround shapes the seizure spread (Schevon et al., 2012). Cortical seizures propagate along the neural pathways but with large temporal variability that depends on local inhibitory tone (Wenzel et al., 2017). Also, frontal lobe seizure spread through the basal ganglia, where 95% of the striatal medium spiny neurons are GABAergic^23^. Some seizures required several hundred milliseconds to arrive at the striatum or substantia nigra reticulata, whereas others took seconds to appear in those structures, suggesting stochastic spread based on latency (Brodovskaya et al., 2021). Internal inhibition within these structures might prevent immediate ictal onset within them.

The standard consolidation model proposes that the hippocampus initially stores memory traces that later fade from the hippocampus and get transferred to the neocortical modules, becoming hippocampus-independent (Frankland and Bontempi, 2005; Tse et al., 2007; Kesner and Rolls, 2015; Squire et al., 2015; Barry and Maguire, 2019). Selective cell activity that begins in the hippocampus entrains other brain regions in the cortex, with seizures disrupting this pattern. Previous studies reported the CA1 engram for spatial memory (Naik et al., 2021). Other studies have also reported that the hippocampal engram in the CA1 maps experiences (Choi et al., 2018; Tanaka, 2021; Tanaka and McHugh, 2018). An important hippocampal subfield, the DG separates patterns and plays a role in memory storage (Liu et al., 2012; Squire et al., 2015; Tonegawa et al., 2015; Josselyn and Tonegawa, 2020). Like CA1, DG activation was transient on day 3, suggesting the memory trace has become hippocampus independent. Hippocampal and neocortical coupling during consolidation is primarily derived from electrophysiological recordings of sharp-wave ripples, spindle oscillations, and θ-γ coupling present during encoding or sleep (Buzsáki, 2015; Joo and Frank, 2018). However, direct visualization of neuronal ensembles following learning and consolidation remained challenging until the activity-dependent IEG reporter mice were generated (Guenthner et al., 2013; Josselyn and Tonegawa, 2020). We report a map of spatial memory-activated neurons that spans beyond the hippocampal CA1 in the mPFC, MD, and RSC.

The mPFC connects with the dorsal hippocampus and is an important hub in the consolidation process. mPFC role during recent acquisitions and retrieval of remote memories is supported by both human and nonhuman studies (Gelinas et al., 2016; Tanaka, 2021). We find mPFC was already active during early learning (on day 2 of training), contrary to the standard consolidation theory, which proposes delayed recruitment of cortical ensembles. However, recent studies have also found that fear engrams appear early in cortical areas (Josselyn and Tonegawa, 2020). Surprisingly, unlike CA1 and DG, where we observed a drop in tagged neuronal ensembles on day 3, mPFC was as active on day 3 as on the second day of training. The mPFC neuronal ensembles may facilitate successful performance and accuracy in mice on day 3.

We report similar activation dynamics in the RSC, which was significantly engaged on day 2 of training. The RSC, like PFC, was re-engaged on day 3 supporting that cortical modules are active during recent retrieval. Previous studies have shown that in addition to the presence of head direction cells in RSC, it acts as an interface between hippocampal formation and cortical sensory regions, supporting its crucial role for encoding and retrieving spatial memory (Sugar et al., 2011; Buzsáki, 2015). We also found activation of MD nucleus during early learning on day 2 and retrieval on day 3 of the T-maze task. This co-activation is not surprising since MD has reciprocal connections with the mPFC and exhibits minimal connectivity with either motor or sensory pathways (Mitchell and Chakraborty, 2013). Our study supports the role of MD in the neuronal network activated by spatial memory. In contrast to MD, the tagged neuronal population in the PVT remained unchanged with learning, supporting that the tagged neuronal ensembles specifically in structures, i.e., DG, RSC, mPFC, MD, and CA1, are a part of the neuronal network complex for the memory trace. Wide representation of spatial learning-activated neurons reported here is similar to the fear memory studies that demonstrate that aversive learning is also widely distributed in the brain (Joo and Frank, 2018).

Because the T-maze spatial working memory task depends on the GluA1 subunit plasticity of AMPA receptors (Sanderson et al., 2008), we used mice lacking the GluA1 subunit, who have deficits in hippocampus-dependent spatial working memory tasks but intact spatial reference memory (Reisel et al., 2002; Sanderson et al., 2008). We bred GluA1 KO with TRAP2 mice to validate that specific neuronal ensemble represents the memory trace. These mice failed to learn rewarded alternation task and showed a hyperactive-phenotype as reported previously (Reisel et al., 2002; Sanderson et al., 2008). They did not have learning-tagged neuronal ensembles in any learning-associated brain structures, CA1, DG, mPFC, RSC, and MD. However, the PVT region was as active as in the control TRAP2 mice based on the presence of tagged neurons. Although the absence of GluA1 may increase neuronal activation threshold, previous studies determined that global removal of the GluA1 subunit did not affect PTZ seizure threshold or susceptibility (Adotevi et al., 2020). Loss of plasticity mechanisms disrupted the learning and consolidation patterns.

We used the dual-labeling technique and looked for the overlap between memory and seizure-activated neurons. There was a greater overlap in cortical areas, where RSC showed a 33% and mPFC 35% overlap among the memory and seizure-tagged neurons, exceeding the probability levels for double labeling (0.23% and 0.070%, respectively; Keller et al., 2018; Murakami et al., 2018). An overlap of this level in RSC and mPFC suggests that a seizure would erode the learning ensemble information postacquisition, resulting in memory impairment during recent retrieval. However, the hippocampal subfield CA1 showed 11% overlap, whereas only 2% of tagged neurons overlapped in the DG (with 0.0018% and 0.0011% probability overlaps, respectively; Keller et al., 2018). The absence of any overlap in the PVT nucleus, otherwise active during both experiences, also supports its limited role in spatial memory neuronal ensemble.

The DG overlap was less than the overlap in other brain regions. This likely is because of the sparse coding function of the DG (Piatti et al., 2013). Previous studies demonstrated that activity levels in the DG are lower than in the upstream cortical areas (Piatti et al., 2013). DGCs receive inputs from a smaller population in the EC, and its representation is thought to be expanded onto the larger population of DGCs (Piatti et al., 2013). The basal activation of DGCs is estimated to be 1–2% of the total population, similar to our findings (Piatti et al., 2013). Sparse coding in the DG could also be one of the reasons for the lack of overlap between task learning-activated and seizure-activated cells. Neural activity in the DG is sparse both in terms of the proportion of active neurons and their mean firing rates (Piatti et al., 2013). Studies that mapped neuronal activation during novel environment exploration, however, found that the DG exhibited significantly more activated neurons when compared with the familiar (Mazurkiewicz et al., 2022). Therefore, different types of memories can activate different circuits. It is possible that seizures also interfere with encoding a novel environmental context.

In summary, we demonstrate that seizure and memory occupy the same space in the brain. Increased plasticity, more active pathways, or a combination of both, increased the probability of cells being recruited by a seizure. Seizure and memory consolidation pathways overlapped in the neuronal network of extrahippocampal structures, which may explain seizure-induced retrograde amnesia.

## Synthesis

Reviewing Editor: Viji Santhakumar, University of California Riverside Decisions are customarily a result of the Reviewing Editor and the peer reviewers coming together and discussing their recommendations until a consensus is reached. When revisions are invited, a fact-based synthesis statement explaining their decision and outlining what is needed to prepare a revision will be listed below. The following reviewer(s) agreed to reveal their identity: Laura Ewell, Jeremy Barry.

The manuscript presents an elegant study examining neuronal activation in multiple brain regions involved in learning and consolidation of an alternation task. The simple approach provides insights into the time course and distribution of cell activation patterns during the learning and consolidation process. Careful control experiments confirm GluA1 dependence of task-related neuronal activation. They then show that the same circuits engaged by the alternation task are also activated during an acute chemically induced seizure. By co-labeling for task-related TRAP cells and Arc expression after a chemical seizure, they show that cortical but not hippocampal structures show overlap between task and seizure-activated neurons. The findings are novel and interesting. Some additional clarification would strengthen the study.

1. While the difference between regions is compelling, the possibility that the overlap reflects an expected probability of overlap when labeling large cohorts of neurons cannot be ruled out. Since authors know the number of cells activated by memory - and the number of cells activated by seizure - it should be possible to calculate a joint probability of overlap? It would be compelling to demonstrate that the probability of colabeling observed experimentally exceeds that expected by chance. Such an analysis would strengthen the conclusion that seizures hijack relevant memory ensembles.

2. In a related issue, can the lack of overlap between task and seizure activated DG neurons be attributed to sparse coding in the DG? This is an important finding, and the details concerning one blade of the dentate being more active than another is intriguing. In many respects, DG cell activation is fundamentally unlike the cortex and is sparse by design. Is it then possible to statistically compare cell activation in DG to other regions? While the lack of overlap is still significant, authors should discuss why recently recruited DG cells in task learning might be less likely to be recruited in a seizure?

3. Although the GluA1 experiment is a valid control, it is possible that GluA1 could increase threshold for cell activation. This needs to be discussed. In this regard, the authors could consider an additional control group exposed to a novel environment. The cell recruitment encoding a novel environmental context could be compared to the pattern recruited to a working memory task.

4. Day 2 of alternation training is hard to characterize. Statistically, it isn’t “learning” only day 3 is significantly greater than baseline at day 1. However, it is clear that from a physiological point of view, day 2 is where the activity patterns are created, and then set on day 3. This issue should be discussed.

5. There are some minor language and terminology issues, most of which speak more to our poor understanding of consolidation. For example, it may be too colloquial to describe consolidation as memories “moving” from one region to another or being “hijacked” by seizures. Rather, there is pattern of selective cell activity that begins in the hippocampus which entrains other brain regions in the cortex. Seizures perturb or disrupt this selective activity pattern. While this is a matter of clarity, other statements like seizures “causing retrograde amnesia” are more complex. It would be sufficient for the authors to state that the loss of this selective activation of cortical cells correlates with retrograde amnesia as described in other referenced experiments. Loss of plasticity mechanisms is shown here to also disrupt the learning and consolidation patterns in this experiment.

6. The claim that seizures “ride an existing pathway to spread” is very important and intriguing idea but may need to be tempered somewhat. Whether specific pathways or general levels of altered plasticity in different brain regions are in play, needs further experimentation. For example, if the authors trained the mice in a task that involved greater RSC than mPFC activation, would there be more seizure activation in RSC? Likewise, if plasticity levels were increased with a pharmacological intervention, would seizure recruitment increase globally? Including the dentate gyrus? Lastly, if a region of the cortex was inactivated during the training of the task, assuming this region wasn’t critical for task learning, and the drug was allowed to wash out before seizure induction, would cells in that region still be recruited by the seizure? While these manipulations are presently beyond the scope of the current manuscript, I authors should consider rephrasing the findings as pointing to increased plasticity or more active pathways, or a combination of both, that increases the probability of cells being recruited by seizure. Another consideration is provided by the Yuste lab (PMID 28658617) where they found that seizures can propagate along the same neural pathways, but that there was a great deal of temporal variability that depended on local inhibitory tone. Might the alternation task be altering inhibitory tone in specific brain regions involved in task performance? As an aside, there are parallels to this idea in glioblastoma research (see PMID 25913192) where tumor generation tends to follow neural activity patterns. Some of these issues could be discussed

7. The journal does not usually allow supplemental materials and the authors should consider including as supp data as party of main text

8. Minor edits:

a. Please clarify “fixed” memory deficits?

b. Seizures “use” the memory circuit the same way a river “uses” a river bed

c. -...reported that “the” hippocampal engram...

d. -...suggesting that “the” memory trace has become...

- Figure 1: “dotsrepresent"

e. - Figure 5: “homacage” and insert a cartoon of the homecage

## Author Response

The manuscript presents an elegant study examining neuronal activation in multiple brain regions involved in learning and consolidation of an alternation task. The simple approach provides insights into the time course and distribution of cell activation patterns during the learning and consolidation process. Careful control experiments confirm GluA1 dependence of task-related neuronal activation. They then show that the same circuits engaged by the alternation task are also activated during an acute chemically induced seizure. By co-labeling for task-related TRAP cells and Arc expression after a chemical seizure, they show that cortical but not hippocampal structures show overlap between task and seizure-activated neurons. The findings are novel and interesting. Some additional clarification would strengthen the study.

We thank the reviewers for their comments. We modified our manuscript accordingly and highlighted changes in yellow.

1. While the difference between regions is compelling, the possibility that the overlap reflects an expected probability of overlap when labeling large cohorts of neurons cannot be ruled out. Since authors know the number of cells activated by memory - and the number of cells activated by seizure - it should be possible to calculate a joint probability of overlap? It would be compelling to demonstrate that the probability of colabeling observed experimentally exceeds that expected by chance. Such an analysis would strengthen the conclusion that seizures hijack relevant memory ensembles.

We have performed these calculations and included them in the revised manuscript and Methods.

According to the previous publication1: joint probability of overlap calculated as ((cFos+/Total DAPI+) x (CTB555+/Total DAPI+)) x 100%.

In our case: overlap probability = ((Day 2 learning-activated tdT+ve neurons in RSC/Total DAPI+ve neurons in RSC) x (seizure-activated tdT+ve neurons in RSC/Total DAPI+ve neurons in RSC)) x 100 probability of overlap, RSC = ((3466/98,148) x (6560/98,148)) x 100% = 0.23% observed overlap, RSC = 33% (Neuron density in RSC = 98,148 (Murakami et al., 2018, Keller et al., 2018).

Chance overlap, mPFC = ((3127/121,000) x (3255/121,000)) x 100% = 0.070% obsereved overlap, mPFC = 35% (Cell density for mPFC is the frontal cortex (ave. 121,000)) overlap likelihood, CA1 = ((258/65,000) x (293/65,000)) x 100% = 0.0018% observed overlap, CA1 = 11% overlap probability, DG = ((859/501,240) x (3091/501,240)) x 100% = 0.0011% observed overlap, DG = 2%

The observed overlaps are much higher than the probability of random overlaps.

2. In a related issue, can the lack of overlap between task and seizure activated DG neurons be attributed to sparse coding in the DG? This is an important finding, and the details concerning one blade of the dentate being more active than another is intriguing. In many respects, DG cell activation is fundamentally unlike the cortex and is sparse by design. Is it then possible to statistically compare cell activation in DG to other regions? While the lack of overlap is still significant, authors should discuss why recently recruited DG cells in task learning might be less likely to be recruited in a seizure?

We note that the DG overlap was less than the overlap in other regions. This likely is due to the sparse coding function of DG. Previous studies demonstrated that activity levels in the DG are lower than in the upstream cortical areas4. DGCs receive inputs from a smaller population in the entorhinal cortex, and its representation is thought to be expanded onto the larger population of DGCs. The basal activation of DGCs is estimated to be 1-2% of the total population4, similar to our findings.

Sparse coding in the DG could also be one of the reasons why we find the lack of overlap between task learning- and seizure-activated cells. Neural activity in the DG is sparse both in terms of the proportion of active neurons and their mean firing rates. We have included a new paragraph at the end of the Discussion.

3. Although the GluA1 experiment is a valid control, it is possible that GluA1 could increase threshold for cell activation. This needs to be discussed. In this regard, the authors could consider an additional control group exposed to a novel environment. The cell recruitment encoding a novel environmental context could be compared to the pattern recruited to a working memory task.

We agree that absence of GluA1 may increase neuronal activation threshold. We note this limitation in the discussion; however, previous studies determined that global removal of the GluA1 subunit did not affect PTZ seizure threshold or susceptibility.

A different research group has already mapped neuronal activation in TRAP2 mice exposed to a novel environment5. The DG exhibited significantly more activated neurons during novel environment exploration when compared to the familiar. Several other regions (primary/secondary motor cortex, PLC, ILC, visual cortex, CA1, CA3) exhibited only modest increases in neuronal activation. Therefore, different types of memories might activate different circuits. It is possible that seizures also interfere with encoding a novel environmental context. We have included this at the end of the Discussion.

4. Day 2 of alternation training is hard to characterize. Statistically, it isn’t “learning” only day 3 is significantly greater than baseline at day 1. However, it is clear that from a physiological point of view, day 2 is where the activity patterns are created, and then set on day 3. This issue should be discussed.

We agree that memory encoding occurs on Day 2, and memory retrieval occurs on Day 3. We have corrected the manuscript.

5. There are some minor language and terminology issues, most of which speak more to our poor understanding of consolidation. For example, it may be too colloquial to describe consolidation as memories “moving” from one region to another or being “hijacked” by seizures. Rather, there is pattern of selective cell activity that begins in the hippocampus which entrains other brain regions in the cortex. Seizures perturb or disrupt this selective activity pattern. While this is a matter of clarity, other statements like seizures “causing retrograde amnesia” are more complex. It would be sufficient for the authors to state that the loss of this selective activation of cortical cells correlates with retrograde amnesia as described in other referenced experiments. Loss of plasticity mechanisms is shown here to also disrupt the learning and consolidation patterns in this experiment.

We agree that a pattern of selective cell activity in the hippocampus entrains other cortical brain regions, and seizures disrupt this selective activity pattern. We have revised the manuscript and tried to use more precise terminology.

It is well established in the literature, however, that seizures cause retrograde amnesia, and patients with seizures experience memory problems. For example, transient epileptic amnesia is a clinical presentation of patients with temporal lobe epilepsy.

We agree that loss of plasticity mechanisms disrupts the learning and consolidation patterns. We included a new paragraph in the Discussion.

6. The claim that seizures “ride an existing pathway to spread” is very important and intriguing idea but may need to be tempered somewhat. Whether specific pathways or general levels of altered plasticity in different brain regions are in play, needs further experimentation. For example, if the authors trained the mice in a task that involved greater RSC than mPFC activation, would there be more seizure activation in RSC? Likewise, if plasticity levels were increased with a pharmacological intervention, would seizure recruitment increase globally? Including the dentate gyrus? Lastly, if a region of the cortex was inactivated during the training of the task, assuming this region wasn’t critical for task learning, and the drug was allowed to wash out before seizure induction, would cells in that region still be recruited by the seizure? While these manipulations are presently beyond the scope of the current manuscript, I authors should consider rephrasing the findings as pointing to increased plasticity or more active pathways, or a combination of both, that increases the probability of cells being recruited by seizure. Another consideration is provided by the Yuste lab (PMID 28658617) where they found that seizures can propagate along the same neural pathways, but that there was a great deal of temporal variability that depended on local inhibitory tone. Might the alternation task be altering inhibitory tone in specific brain regions involved in task performance? As an aside, there are parallels to this idea in glioblastoma research (see PMID 25913192) where tumor generation tends to follow neural activity patterns. Some of these issues could be discussed

We agree that anatomical connectivity, together with neuronal excitability, inhibitory surround, and neuronal plasticity, shape the seizure circuit. The pattern of neuronal activation during seizures might be a stochastic process guided by the increased plasticity of certain cells. Our current ongoing experiments will address some of these questions. Our previous work demonstrated that global removal of the GluA1 subunit of AMPA receptor reduced susceptibility to status epilepticus. GluA1 knockout reduced mortality, severity, and duration of status epilepticus. This indicates that multiple plasticity mechanisms are critical for the occurrence and maintenance of seizures. We included this in the manuscript’s Discussion.

We also agree that the inhibitory tone might also play a role in shaping the seizure circuit. Our previous findings support this idea7. We have demonstrated that neuronal firing and synchrony are more correlated along the lamellar axis in the hippocampus during seizures that contains excitatory connections than along the septotemporal axis that has inhibitory connections. Also, studies in the hippocampus and neocortex demonstrate a strong role for inhibition in shaping seizure spread. Propagation of seizures through the hippocampus is gated by strong inhibition of the DG9. Similarly, in the neocortex, the inhibitory surround shapes the seizure spread10. We have also observed that during the spread of frontal lobe seizures through the basal ganglia, some seizures required several hundred milliseconds to arrive at the striatum or SNR, whereas other seizures took several seconds (up to 10 s) to appear in those structures, suggesting stochastic spread based on latency. Internal inhibition within these structures during some seizures might prevent immediate ictal onset within them.

7. The journal does not usually allow supplemental materials and the authors should consider including as supp data as party of main text

We changed it to Figure 6.

8. Minor edits:

a. Please clarify “fixed” memory deficits?

The paper we cite by Gelinas et al. specifies: “This pathological hippocampal-cortical coupling may impair cognition in individuals with temporal lobe epilepsy", so we fixed the sentence accordingly.

b. Seizures “use” the memory circuit the same way a river “uses” a river bed

c. -...reported that “the” hippocampal engram...

d. -...suggesting that “the” memory trace has become...

- Figure 1: “dotsrepresent"

e. - Figure 5: “homacage” and insert a cartoon of the homecage

A “homecage” is a standard laboratory mouse cage (12x6x5 in) filled with corn cob bedding, so we believe including its picture is redundant. However, we included the description of the homecage and its dimensions in the Methods (p. 4). We have also made the rest of the corrections, thank you.

References

1. Roy, D.S. et al. Distinct neural circuits for the formation and retrieval of episodic memories. Cell. 170, (2017).

2. Keller, D. et al. Cell densities in the mouse brain: a systematic review. Front Neuroanat. 12, (2018).

3. Murakami, T.C. et al. A three-dimensional single-cell-resolution whole-brain atlas using CUBIC-X expansion microscopy and tissue clearing. Nat. Neurosci. 21, (2018).

4. Piatti, V.C, Ewell, L.A., Leutgeb, J.K. Neurogenesis in the dentate gyrus: carrying the message or dictating the tone. Front. Neurosci. 7, (2013).

5. Mazurkiewicz, M., et. al. Neuronal activity mapping during exploration of a novel environment. Brain Res. 1776, (2022).

6. Sanderson, D.J., et al. Spatial working memory deficits in GluA1 AMPA receptor subunit knockout mice reflect impaired short-term habituation: Evidence for Wagner’s dual-process memory model. Neuropsychologia. 48, 2303-2315 (2010).

7. Ren, X., Brodovskaya, A., Hudson, J. L. & Kapur, J. Connectivity and neuronal synchrony during seizures. J. Neurosci. 41, 7623-7635 (2021).

8. Sloviter, R.S, Lømo T. Updating the lamellar hypothesis of hippocampal organization. Front Neural Circuits. 6, (2012).

9. Krook-Magnuson, E. et al. In vivo evaluation of the dentate gate theory in epilepsy. J Physiol. 593, 2379-2388 (2015).

10. Schevon, C. A. et al. Evidence of an inhibitory restraint of seizure activity in humans. Nat. Commun. 3, (2012).

11. Brodovskaya, A, Shiono, S, Kapur, J. Activation of the basal ganglia and indirect pathway neurons during frontal lobe seizures. Brain. 144, (2021).

12. Brodovskaya, A. & Batabyal, T. et al. Distinct roles of rodent thalamus and corpus callosum in seizure generalization. Ann Neurol. 91, (2022).
